# Crystal structure and Hirshfeld surface analysis of (*Z*)-2-amino-4-(2,6-di­chloro­phen­yl)-5-(1-hy­droxy­ethyl­idene)-6-oxo-1-phenyl-1,4,5,6-tetra­hydro­pyridine-3-carbo­nitrile

**DOI:** 10.1107/S2056989021007994

**Published:** 2021-08-10

**Authors:** Farid N. Naghiyev, Anastasiya V. Pavlova, Victor N. Khrustalev, Mehmet Akkurt, Ali N. Khalilov, Anzurat A. Akobirshoeva, İbrahim G. Mamedov

**Affiliations:** a Department of Chemistry, Baku State University, Z. Khalilov str. 23, Az, 1148 Baku, Azerbaijan; b Peoples’ Friendship University of Russia (RUDN University), Miklukho-Maklay St. 6, Moscow, 117198 , Russian Federation; c N. D. Zelinsky Institute of Organic Chemistry RAS, Leninsky Prosp. 47, Moscow, 119991, Russian Federation; d Department of Physics, Faculty of Sciences, Erciyes University, 38039 Kayseri, Turkey; e "Composite Materials" Scientific Research Center, Azerbaijan State Economic University (UNEC), H. Aliyev str. 135, Az 1063, Baku, Azerbaijan; f Acad. Sci. Republ. Tadzhikistan, Kh. Yu. Yusufbekov Pamir Biol. Inst., 1 Kholdorova St, Khorog 736002, Gbao, Tajikistan

**Keywords:** crystal structure, pyridine ring, hydrogen bonds, ring motifs, Hirshfeld surface analysis

## Abstract

The mol­ecular conformation of the title compound, C_20_H_15_Cl_2_N_3_O_2_, is stabilized by an intra­molecular O—H⋯O hydrogen bond, forming an *S*(6) ring motif. The central pyridine ring is almost planar [maximum deviation = 0.074 (3) Å]. It subtends dihedral angles of 86.10 (15) and 87.17 (14)°, respectively, with the phenyl and di­chloro­phenyl rings, which are at an angle of 21.28 (15)° to each other. The =C(—OH)CH_3_ group is coplanar. In the crystal, mol­ecules are linked by inter­molecular N—H⋯N and C—H⋯N hydrogen bonds, and N—H⋯π and C—H⋯π inter­actions, forming a three-dimensional network. The most important contributions to the crystal packing are from H⋯H (33.1%), C⋯H/H⋯C (22.5%), Cl⋯H/H⋯Cl (14.1%), O⋯H/H⋯O (11.9%) and N⋯H/H⋯N (9.7%) inter­actions.

## Abstract 2

The mol­ecular conformation of the title compound is stabilized by an intra­molecular O—H⋯O hydrogen bond, forming an *S*(6) ring motif. Inter­molecular N—H⋯N and C—H⋯N hydrogen bonds, as well as N—H⋯π and C—H⋯π inter­actions create a three-dimensional network in the crystal.

## Chemical context   

The development of effective methods for the construction of small-sized mol­ecules bearing a nitro­gen heterocycle is a very important proposition in organic synthesis and catalysis (Abdel-Hafiz *et al.*, 2012 ▸; Gurbanov *et al.*, 2018 ▸; Zubkov *et al.*, 2018 ▸). As members of this family, pyridine derivatives play a key role in flavor chemistry, crystal engineering, and the development of biologically active compounds (Adams & De Kimpe, 2006 ▸; Mahmoudi *et al.*, 2019 ▸; Mamedov *et al.*, 2020 ▸). The pyridine core is a key bioactive fragment of diverse natural products (niacin, pyridoxine, nicotine, NADP^+^) and series of derivatives constitute promising drugs in medicinal chemistry (Mohsin & Ahmad, 2018 ▸).
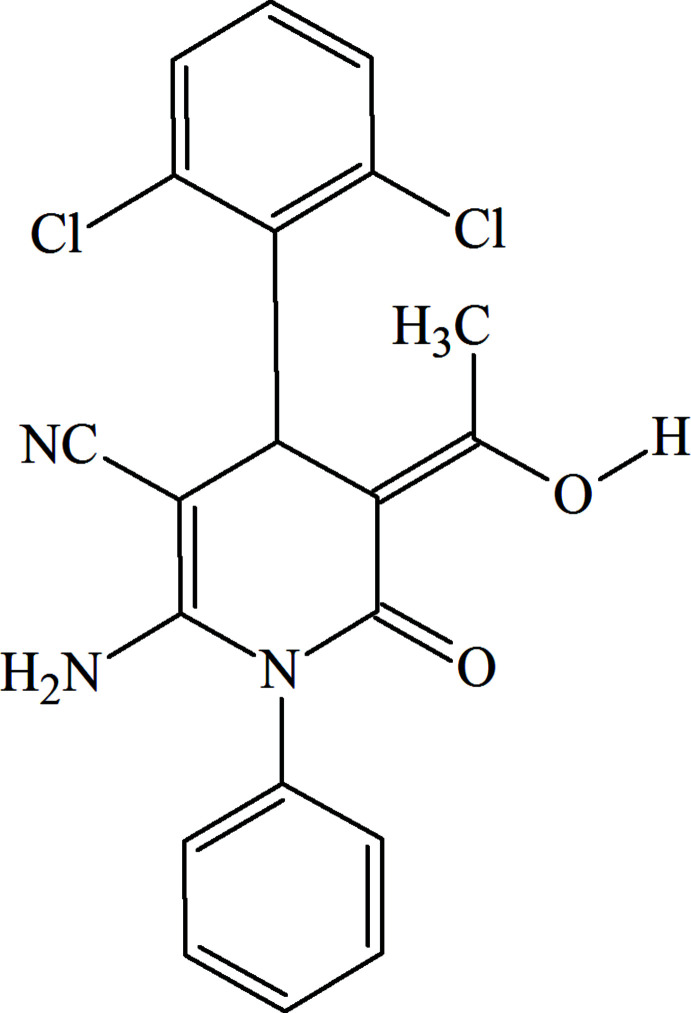


In this study, in the framework of our ongoing structural studies (Naghiyev *et al.*, 2020 ▸, 2021*a* ▸,*b* ▸), we report the crystal structure and Hirshfeld surface analysis of the title compound, (*Z*)-2-amino-4-(2,6-di­chloro­phen­yl)-5-(1-hy­droxy­ethyl­idene)-6-oxo-1-phenyl-1,4,5,6-tetra­hydro­pyridine-3-carbo­nitrile, prev­iously mistakenly reported in the *E* isomeric form (Maharramov *et al.*, 2018 ▸). This compound was also previously mentioned as transient inter­mediate but neither isolated nor characterized (Naghiyeva *et al.*, 2019 ▸).

## Structural commentary   

**Figure 1:**
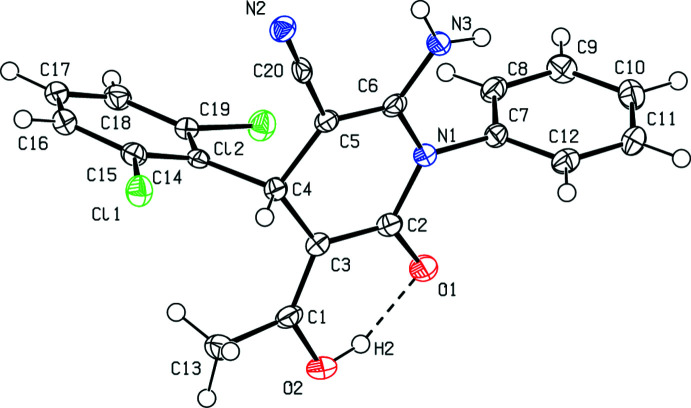
The mol­ecular structure of the title compound showing the atom-numbering scheme and displacement ellipsoids at the 50% probability level.

The title compound crystallizes in the monoclinic space group *P*2_1_/*c* with *Z* = 4, in which the asymmetric unit comprises one mol­ecule. In the mol­ecule (Fig. 1 ▸), the central pyridine ring (N1/C2–C6) is almost planar with a maximum deviation of 0.074 (3) Å for C4. The phenyl (C7–C12) and di­chloro­phenyl (C14–C19) rings are at an angle of 21.28 (15)°. They form dihedral angles of 86.10 (15) and 87.17 (14)°, respectively, with the central pyridine ring. The =C(—OH)CH_3_ group is nearly coplanar with the pyridine ring with C2—C3—C1—O2 and C4—C3—C1—C13 torsion angles of only 5.5 (5) and 3.3 (5)°, respectively. A strong intra­molecular O2—H2⋯O1 hydrogen bond (Fig. 1 ▸, Table 1 ▸) stabilizes the mol­ecular conformation of the title mol­ecule, creating an *S*(6) ring motif (Bernstein *et al.*, 1995 ▸).

## Supra­molecular features and Hirshfeld surface analysis   

**Figure 2:**
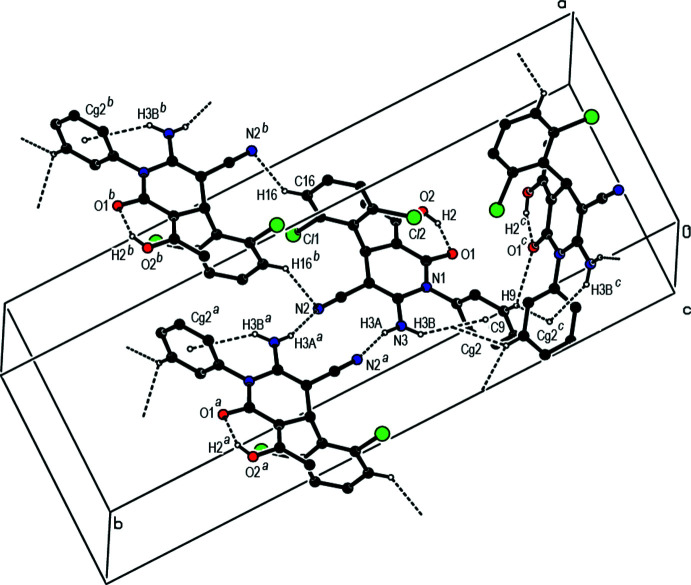
A general view of the intra- and inter­molecular O—H⋯O, N—H⋯N hydrogen bonding and N—H⋯π and C—H⋯π inter­actions in the title compound. Symmetry codes: (*a*) 1 − *x*, 1 − *y*, 2 − *z*; (*b*) 2 − *x*, 1 − *y*, 2 − *z*; (*c*) *x*, 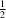 − *y*, 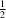 + *z*.

Inter­molecular N3—H3*A*⋯N2 hydrogen bonds, which form an 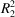
(12) ring motif between pairs of mol­ecules along the *b-*axis direction and an 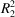
(16) ring motif between pairs of mol­ecules along the *a*-axis direction, together with N3—H3*B*⋯*Cg*2 and C9—H9⋯*Cg*2 inter­actions (Fig. 2 ▸, Tables 1 ▸ and 2 ▸; *Cg*2 is the centroid of the C7–C12 phenyl ring) create a three-dimensional network in the crystal (Figs. 2 ▸ and 3 ▸).

**Figure 4:**
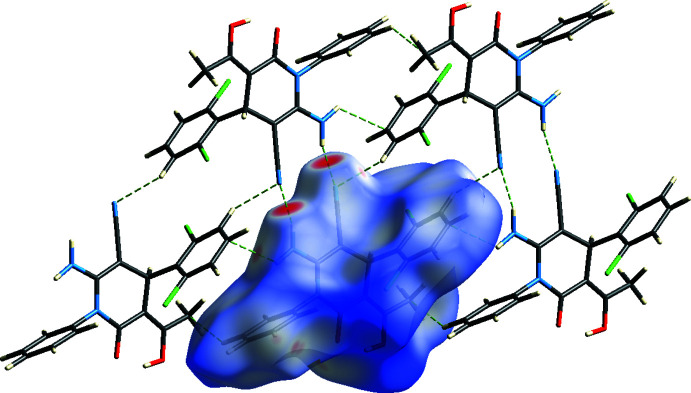
Hirshfeld surface of the title compound mapped with *d*_norm_.

**Figure 5:**
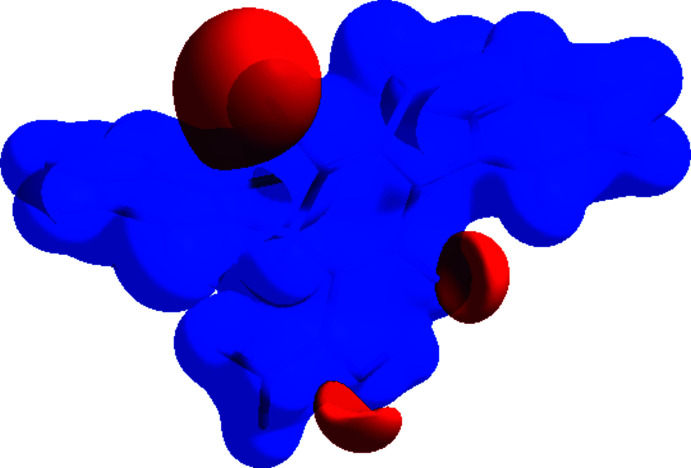
View of the three-dimensional Hirshfeld surface of the title compound plotted over electrostatic potential energy in the range −0.0500 to 0.0500 a.u. using the STO-3 G basis set at the Hartree–Fock level of theory. Hydrogen-bond donors and acceptors are shown as blue and red regions, respectively, around the atoms, corresponding to positive and negative potentials.

The Hirshfeld surfaces were calculated and the two-dimensional fingerprint plots generated using *Crystal Explorer 17.5* (Turner *et al.*, 2017 ▸). The use of various hues and intensities to represent short and long contacts, as well as the relative intensity of the connections, allows Hirshfeld surfaces to depict inter­molecular inter­actions. Fig. 4 ▸ shows the three-dimensional Hirshfeld surfaces of the title compound plotted over *d*_norm_ (normalized contact distance) in the range of −0.4290 to 1.5192 a.u. The red patches that appear around N2 are caused by the inter­molecular N3—H3*A*⋯N2 and C16—H16⋯N2 inter­actions, which are important in the packing of the title mol­ecule. Bright red dots near N2 and amine hydrogen atoms H3*A* and H3*B* highlight their functions as hydrogen-bonding acceptors and donors, respectively; these also appear as blue and red areas on the Hirshfeld surface mapped over electrostatic potential (Spackman *et al.*, 2008 ▸) in Fig. 5 ▸, corresponding to positive and negative potentials. Positive electrostatic potential (hydrogen-bond donors) is shown in blue, whereas negative electrostatic potential is indicated in red (hydrogen-bond acceptors).

**Figure 6:**
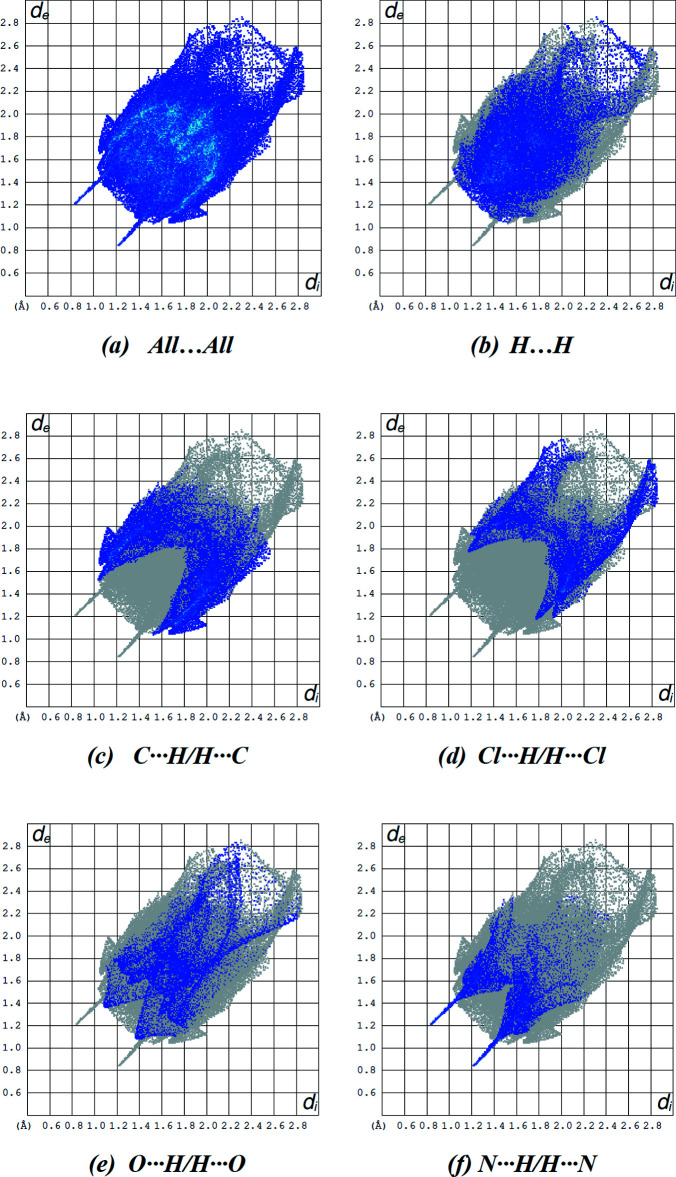
The two-dimensional fingerprint plots of the title compound, showing (*a*) all inter­actions, and delineated into (*b*) H⋯H, (*c*) C⋯H/H⋯C, (*d*) Cl⋯H/H⋯Cl, (*e*) O⋯H/H⋯O and (*f*) N⋯H/H⋯N inter­actions [*d*_e_ and *d*_i_ represent the distances from a point on the Hirshfeld surface to the nearest atoms outside (external) and inside (inter­nal) the surface, respectively].

In Fig. 6 ▸, the overall two-dimensional fingerprint plot for the title compound and those delineated into H⋯H, C⋯H/H⋯C, Cl⋯H/H⋯Cl, O⋯H/H⋯O and N⋯H/H⋯N contacts, as well as their relative contributions to the Hirshfeld surface, are presented, while details of the various contacts are given in Table 2 ▸. The percentage contributions to the Hirshfeld surfaces from the various inter­atomic contacts are as follows: H⋯H (33.1%; Fig. 6 ▸ *b*), C⋯H/H⋯C (22.5%; Fig. 6 ▸ *c*), Cl⋯H/H⋯Cl (14.1%; Fig. 6 ▸ *d*), O⋯H/H⋯O (11.9%; Fig. 6 ▸ *e*) and N⋯H/H⋯N (9.7%; Fig. 6 ▸ *f*). Other Cl⋯C/C⋯Cl, C⋯C, Cl⋯O/O⋯Cl, Cl⋯N/N⋯Cl, N⋯C/C⋯N, O⋯N/N⋯O, Cl⋯Cl, O⋯C/C⋯O and N⋯N contacts contribute less than 2.1% to Hirshfeld surface mapping and have little directional influence on mol­ecular packing (Table 3 ▸).

## Database survey   

A search of the Cambridge Structural Database (CSD, Version 5.39, update of August 2018; Groom *et al.*, 2016 ▸) using *Conquest* (Bruno *et al.*, 2002 ▸) for the tetra­hydro­pyridine unit revealed 1339 hits. Some inter­esting structures related to the title compound based on their tetra­hydro­pyridine moieties include: ethyl 4-hy­droxy-2,6-diphenyl-5-(phenyl­sulfan­yl)pyri­dine-3-carboxyl­ate (refcode SETWOE: Suresh *et al.*, 2007 ▸), ethyl 2,6-bis­(4-fluoro­phen­yl)-4-hy­droxy-5-(4-methyl­phenyl­sulfan­yl)pyridine-3-carboxyl­ate (SETWUK: Suresh *et al.*, 2007 ▸), 2,6-di­amino-4-chloro­pyrimidin-1-ium 2-carb­oxy-3-nitro­benzoate (JEBRAM: Mohana *et al.*, 2017 ▸) and 2,6-di­amino-4-chloro­pyrimidin-1-ium 4-methyl­benzene-1-sulfonate monohydrate (JEBREQ: Mohana *et al.*, 2017 ▸).

The polysubstituted pyridines, SETWOE (space group: *P*2_1_/*c*) and SETWUK (space group: *P*2_1_/*n*), adopt nearly planar structures. The crystal structure of SETWOE is stabil­ized by inter­molecular C—H⋯O and C—H⋯π inter­actions. The C—H⋯O hydrogen bonds generate rings with *R^2^_2_* (14) and *R^2^_2_* (20) motifs. The crystal structure of SETWUK is stabilized by inter­molecular C—H⋯F and C—H⋯π inter­actions. The C—H⋯F bond generates a linear chain with a *C*(14) motif. In addition, in SETWOE and SETWUK, intra­molecular O—H⋯O inter­actions are found, which generate an *S*(6) graph-set motif. No significant ar­yl–aryl or π–π inter­actions exist in these structures. All this bears some resemblance to the title compound.

In both the related salts, JEBRAM (space group: *P* 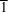) and JEBREQ (space group: *P* 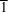) , the N atom in the 1-position of the pyrimidine ring is protonated. In JEBRAM, the protonated N atom and the amino group of the pyrimidinium cation inter­act with the carboxyl­ate group of the anion through N—H⋯O hydrogen bonds, forming a heterosynthon with an *R^2^_2_* (8) ring motif. In the hydrated salt JEBREQ, the presence of the water mol­ecule prevents the formation of the familiar 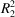
(8) ring motif. Instead, an expanded ring [*i.e. R* ^3^_2_(8)] is formed involving the sulfonate group, the pyrimidinium cation and the water mol­ecule. Both salts form a supra­molecular homosynthon [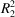
(8) ring motif] through N—H⋯N hydrogen bonds. The mol­ecular structures are further stabilized by π–π stacking, and C=O⋯π, C—H⋯O and C—H⋯Cl inter­actions. None of these are found in the crystal packing of the title compound. It appears that the protonation state of the pyrimidine ring influences the inter­molecular inter­actions within the crystal lattices to a substantial extent.

## Synthesis and crystallization   

The title compound was synthesized using our previously reported procedure (Maharramov *et al.*, 2018 ▸), and colorless prisms were obtained upon recrystallization from its methanol solution.

## Refinement   

Crystal data, data collection and structure refinement details are summarized in Table 4 ▸. The positional parameters of the H atoms of the hy­droxy and amine groups were determined from difference electron-density maps and were refined freely [O2—H2 = 0.86 (4) Å, N3—H3*A* = 0.86 (4) Å and N3—H3*B* = 0.88 (4) Å]. Their isotropic displacement parameters were refined using a riding model with *U*_iso_(H) set to either 1.2*U*_eq_(N) for the NH_2_ group or 1.5*U*_eq_(O) for the OH group. The C-bound H atoms were positioned geometrically (C—H = 0.95–1.00 Å) and allowed to ride on their parent atoms, with *U*_iso_(H) = 1.5*U*_eq_(C) for the methyl group and *U*_iso_(H) = 1.2*U*_eq_(C) for aromatic and methine H atoms.

## Supplementary Material

Crystal structure: contains datablock(s) I. DOI: 10.1107/S2056989021007994/yz2010sup1.cif

Structure factors: contains datablock(s) I. DOI: 10.1107/S2056989021007994/yz2010Isup2.hkl

Supporting information file. DOI: 10.1107/S2056989021007994/yz2010Isup3.cml

CCDC reference: 2101203

Additional supporting information:  crystallographic information; 3D view; checkCIF report

## Figures and Tables

**Figure 3:**
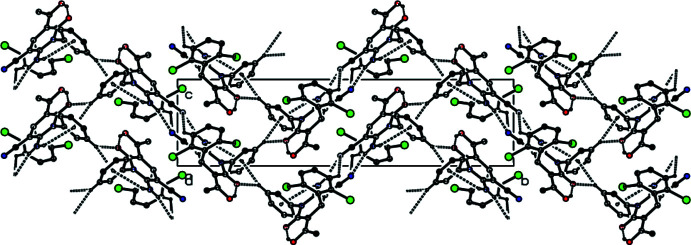
A view down the *a* axis of the crystal packing of the title compound based on the inter­molecular inter­actions shown in Fig. 2 ▸.

**Table 1:** Hydrogen-bond geometry (Å, °) *Cg*2 is the centroid of the C7–C12 phenyl ring.

*D*—H⋯*A*	*D*—H	H⋯*A*	*D*⋯*A*	*D*—H⋯*A*
O2—H2⋯O1	0.86 (4)	1.72 (4)	2.514 (3)	153 (4)
N3—H3*A*⋯N2^i^	0.86 (4)	2.22 (4)	3.032 (4)	159 (3)
C16—H16⋯N2^ii^	0.95	2.62	3.308 (4)	129
N3—H3*B*⋯*Cg*2	0.88 (4)	2.88 (4)	3.581 (3)	138 (3)
C9—H9⋯*Cg*2^iii^	0.95	2.70	3.564 (4)	151

Symmetry codes: (i) -x+1, -y+1, -z+2; (ii) -x+2, -y+1, -z+2; (iii) x, -y-{\script{1\over 2}}, z-{\script{1\over 2}}.

**Table 2:** Inter­atomic contacts of the title compound (Å)

Contact	Distance	Symmetry operation
Cl1⋯Cl1	3.6744 (14)	2 − *x*, 1 − *y*, 1 − *z*
H4⋯C20	2.77	1 − *x*, 1 − *y*, 1 − *z*
O1⋯H9	2.54	*x*, {1\over 2} − *y*, −{1\over 2} + *z*
N2⋯H13*C*	2.81	*x*, *y*, 1 + *z*
H3*A*⋯N2	2.22 (4)	1 − *x*, 1 − *y*, 2 − *z*
H16⋯N2	2.62	2 − *x*, 1 − *y*, 2 − *z*
H11⋯H13*B*	2.54	−1 + *x*, *y*, *z*
H17⋯H3*B*	2.54	1 + *x*, *y*, *z*
H12⋯C18	2.93	−1 + *x*, *y*, −1 + *z*

**Table 3:** Percentage contributions of inter­atomic contacts to the Hirshfeld surface for the title compound

Contact	Percentage contribution
H⋯H	33.1
C⋯H/H⋯C	22.5
Cl⋯H/H⋯Cl	14.1
O⋯H/H⋯O	11.9
N⋯H/H⋯N	9.7
Cl⋯C/C⋯Cl	2.1
C⋯C	1.4
Cl⋯O/O⋯Cl	1.2
Cl⋯N/N⋯Cl	1.1
N⋯C/C⋯N	1.0
O⋯N/N⋯O	0.6
Cl⋯Cl	0.6
O⋯C/C⋯O	0.5
N⋯N	0.1

**Table 4:** Experimental details

Crystal data	
Chemical formula	C_20_H_15_Cl_2_N_3_O_2_
*M*_r_	400.25
Crystal system, space group	Monoclinic, *P*2_1_/*c*
Temperature (K)	100
*a*, *b*, *c* (Å)	9.662 (1), 27.010 (3), 7.4782 (8)
β (°)	111.571 (2)
*V* (Å^3^)	1814.9 (3)
*Z*	4
Radiation type	Mo *K*α
μ (mm^−1^)	0.38
Crystal size (mm)	0.24 × 0.21 × 0.02

Data collection	
Diffractometer	Bruker D8 QUEST PHOTON-III CCD
Absorption correction	Multi-scan (*SADABS*; Krause *et al.*, 2015 ▸)
*T*_min_, *T*_max_	0.864, 0.986
No. of measured, independent and observed [*I* > 2σ(*I*)] reflections	27440, 4180, 2631
*R*_int_	0.099
(sin θ/λ)_max_ (Å^−1^)	0.650

Refinement	
*R*[*F*^2^ > 2σ(*F* ^2^)], *wR*(*F* ^2^), *S*	0.053, 0.126, 1.01
No. of reflections	4180
No. of parameters	255
H-atom treatment	H atoms treated by a mixture of independent and constrained refinement
Δρ_max_, Δρ_min_ (e Å^−3^)	0.50, −0.37

Computer programs: *APEX3* (Bruker, 2018 ▸), *SAINT* (Bruker, 2013 ▸), *SHELXT2014/5* (Sheldrick, 2015*a* ▸), *SHELXL2018/3* (Sheldrick, 2015*b* ▸), *ORTEP-3 for Windows* (Farrugia, 2012 ▸) and *PLATON* (Spek, 2020 ▸).

## supplementary crystallographic information

### Crystal data

**Table:**

C_20_H_15_Cl_2_N_3_O_2_	*F*(000) = 824
*M**_r_* = 400.25	*D*_x_ = 1.465 Mg m^−^^3^
Monoclinic, *P*2_1_/*c*	Mo *K*α radiation, λ = 0.71073 Å
*a* = 9.662 (1) Å	Cell parameters from 2887 reflections
*b* = 27.010 (3) Å	θ = 2.3–25.6°
*c* = 7.4782 (8) Å	µ = 0.38 mm^−^^1^
β = 111.571 (2)°	*T* = 100 K
*V* = 1814.9 (3) Å^3^	Plate, colourless
*Z* = 4	0.24 × 0.21 × 0.02 mm

### Data collection

**Table:**

Bruker D8 QUEST PHOTON-III CCD diffractometer	2631 reflections with *I* > 2σ(*I*)
φ and ω scans	*R*_int_ = 0.099
Absorption correction: multi-scan (SADABS; Krause *et al.*, 2015)	θ_max_ = 27.5°, θ_min_ = 2.3°
*T*_min_ = 0.864, *T*_max_ = 0.986	*h* = −12→12
27440 measured reflections	*k* = −35→35
4180 independent reflections	*l* = −9→9

### Refinement

**Table:**

Refinement on *F*^2^	Secondary atom site location: dual
Least-squares matrix: full	Hydrogen site location: mixed
*R*[*F*^2^ > 2σ(*F*^2^)] = 0.053	H atoms treated by a mixture of independent and constrained refinement
*wR*(*F*^2^) = 0.126	*w* = 1/[σ^2^(*F*_o_^2^) + (0.0387*P*)^2^ + 2.0157*P*] where *P* = (*F*_o_^2^ + 2*F*_c_^2^)/3
*S* = 1.01	(Δ/σ)_max_ < 0.001
4180 reflections	Δρ_max_ = 0.50 e Å^−^^3^
255 parameters	Δρ_min_ = −0.37 e Å^−^^3^
0 restraints	Extinction correction: SHELXL, Fc^*^=kFc[1+0.001xFc^2^λ^3^/sin(2θ)]^-1/4^
Primary atom site location: dual	Extinction coefficient: 0.0024 (2)

### Special details

**Table:**

Geometry. All esds (except the esd in the dihedral angle between two l.s. planes) are estimated using the full covariance matrix. The cell esds are taken into account individually in the estimation of esds in distances, angles and torsion angles; correlations between esds in cell parameters are only used when they are defined by crystal symmetry. An approximate (isotropic) treatment of cell esds is used for estimating esds involving l.s. planes.

### Fractional atomic coordinates and isotropic or equivalent isotropic displacement parameters (Å^2^)

**Table:**

	*x*	*y*	*z*	*U*_iso_*/*U*_eq_	
Cl1	0.86090 (9)	0.51416 (3)	0.60657 (12)	0.0256 (2)	
Cl2	0.73128 (9)	0.32459 (3)	0.75788 (12)	0.0262 (2)	
O1	0.3936 (2)	0.32320 (9)	0.2910 (3)	0.0280 (5)	
O2	0.5668 (3)	0.34472 (9)	0.1210 (3)	0.0286 (6)	
H2	0.502 (4)	0.3295 (15)	0.155 (6)	0.043*	
N1	0.3832 (3)	0.36887 (10)	0.5389 (4)	0.0190 (6)	
N2	0.6028 (3)	0.51570 (10)	0.8819 (4)	0.0213 (6)	
N3	0.3468 (3)	0.41829 (11)	0.7719 (4)	0.0216 (6)	
H3A	0.376 (4)	0.4411 (13)	0.857 (5)	0.026*	
H3B	0.275 (4)	0.3976 (13)	0.763 (5)	0.026*	
C1	0.6193 (3)	0.38087 (12)	0.2516 (4)	0.0222 (7)	
C2	0.4484 (3)	0.35814 (12)	0.4048 (4)	0.0215 (7)	
C3	0.5700 (3)	0.38792 (12)	0.3994 (4)	0.0190 (7)	
C4	0.6393 (3)	0.42841 (12)	0.5454 (4)	0.0185 (7)	
H4	0.6402	0.4592	0.4717	0.022*	
C5	0.5438 (3)	0.43903 (11)	0.6633 (4)	0.0178 (6)	
C6	0.4288 (3)	0.40975 (12)	0.6622 (4)	0.0190 (6)	
C7	0.2636 (3)	0.33754 (11)	0.5457 (4)	0.0182 (6)	
C8	0.2954 (3)	0.29874 (12)	0.6757 (4)	0.0225 (7)	
H8	0.3954	0.2923	0.7572	0.027*	
C9	0.1808 (4)	0.26931 (12)	0.6866 (5)	0.0247 (7)	
H9	0.2018	0.2428	0.7762	0.030*	
C10	0.0355 (4)	0.27893 (12)	0.5655 (5)	0.0254 (7)	
H10	−0.0431	0.2588	0.5722	0.030*	
C11	0.0039 (3)	0.31762 (12)	0.4351 (5)	0.0233 (7)	
H11	−0.0959	0.3239	0.3526	0.028*	
C12	0.1182 (3)	0.34720 (12)	0.4251 (4)	0.0219 (7)	
H12	0.0970	0.3739	0.3362	0.026*	
C13	0.7339 (4)	0.41270 (13)	0.2175 (5)	0.0262 (7)	
H13A	0.7588	0.3988	0.1121	0.039*	
H13B	0.8235	0.4139	0.3346	0.039*	
H13C	0.6943	0.4462	0.1834	0.039*	
C14	0.8003 (3)	0.41904 (12)	0.6829 (4)	0.0168 (6)	
C15	0.9070 (3)	0.45717 (12)	0.7254 (4)	0.0206 (7)	
C16	1.0502 (3)	0.45270 (13)	0.8591 (5)	0.0237 (7)	
H16	1.1177	0.4797	0.8851	0.028*	
C17	1.0934 (3)	0.40785 (13)	0.9546 (4)	0.0252 (7)	
H17	1.1915	0.4041	1.0469	0.030*	
C18	0.9949 (3)	0.36865 (13)	0.9164 (5)	0.0238 (7)	
H18	1.0255	0.3378	0.9793	0.029*	
C19	0.8510 (3)	0.37491 (12)	0.7852 (4)	0.0194 (7)	
C20	0.5773 (3)	0.48144 (12)	0.7841 (4)	0.0190 (7)	

### Atomic displacement parameters (Å^2^)

**Table:**

	*U*^11^	*U*^22^	*U*^33^	*U*^12^	*U*^13^	*U*^23^
Cl1	0.0223 (4)	0.0243 (4)	0.0291 (4)	−0.0013 (3)	0.0083 (3)	0.0025 (4)
Cl2	0.0236 (4)	0.0258 (4)	0.0260 (4)	−0.0006 (3)	0.0056 (3)	0.0049 (4)
O1	0.0271 (12)	0.0342 (14)	0.0240 (12)	−0.0085 (11)	0.0108 (10)	−0.0124 (11)
O2	0.0296 (13)	0.0394 (15)	0.0171 (12)	−0.0043 (11)	0.0091 (10)	−0.0072 (11)
N1	0.0169 (13)	0.0243 (15)	0.0161 (13)	−0.0028 (11)	0.0063 (10)	−0.0035 (11)
N2	0.0213 (14)	0.0210 (14)	0.0200 (14)	−0.0026 (11)	0.0058 (11)	−0.0030 (12)
N3	0.0199 (14)	0.0270 (15)	0.0196 (14)	−0.0071 (12)	0.0095 (11)	−0.0081 (12)
C1	0.0210 (16)	0.0286 (18)	0.0135 (15)	0.0038 (13)	0.0022 (12)	−0.0012 (14)
C2	0.0200 (15)	0.0275 (18)	0.0160 (15)	0.0009 (13)	0.0053 (12)	−0.0023 (14)
C3	0.0174 (15)	0.0225 (17)	0.0152 (15)	−0.0002 (12)	0.0038 (12)	−0.0001 (13)
C4	0.0157 (15)	0.0228 (17)	0.0166 (15)	0.0014 (12)	0.0055 (12)	−0.0020 (13)
C5	0.0172 (15)	0.0207 (16)	0.0139 (15)	0.0005 (12)	0.0037 (12)	−0.0014 (13)
C6	0.0162 (14)	0.0237 (17)	0.0135 (15)	0.0036 (12)	0.0012 (12)	−0.0002 (13)
C7	0.0175 (15)	0.0185 (16)	0.0184 (15)	−0.0037 (12)	0.0062 (12)	−0.0048 (13)
C8	0.0201 (16)	0.0264 (18)	0.0174 (16)	0.0015 (13)	0.0026 (13)	−0.0003 (14)
C9	0.0275 (17)	0.0219 (17)	0.0254 (18)	0.0030 (14)	0.0106 (14)	0.0049 (14)
C10	0.0228 (16)	0.0245 (18)	0.0310 (19)	−0.0028 (14)	0.0124 (14)	−0.0042 (15)
C11	0.0177 (15)	0.0259 (18)	0.0223 (17)	0.0019 (13)	0.0024 (13)	0.0013 (14)
C12	0.0230 (16)	0.0224 (17)	0.0177 (16)	0.0031 (13)	0.0044 (13)	0.0001 (13)
C13	0.0264 (17)	0.033 (2)	0.0206 (17)	0.0037 (15)	0.0107 (14)	0.0041 (15)
C14	0.0152 (14)	0.0241 (16)	0.0126 (14)	0.0025 (12)	0.0070 (11)	−0.0011 (12)
C15	0.0200 (15)	0.0254 (17)	0.0179 (16)	0.0040 (13)	0.0088 (13)	0.0024 (14)
C16	0.0185 (15)	0.0315 (19)	0.0222 (17)	−0.0020 (13)	0.0089 (13)	−0.0035 (14)
C17	0.0162 (15)	0.042 (2)	0.0173 (16)	0.0027 (14)	0.0063 (13)	−0.0010 (15)
C18	0.0219 (16)	0.0307 (19)	0.0196 (16)	0.0047 (14)	0.0087 (13)	0.0047 (14)
C19	0.0174 (15)	0.0266 (17)	0.0150 (15)	−0.0009 (13)	0.0066 (12)	−0.0022 (13)
C20	0.0110 (14)	0.0270 (18)	0.0193 (16)	0.0044 (12)	0.0058 (12)	0.0062 (14)

### Geometric parameters (Å, º)

**Table:**

Cl1—C15	1.751 (3)	C7—C12	1.387 (4)
Cl2—C19	1.747 (3)	C8—C9	1.390 (4)
O1—C2	1.250 (4)	C8—H8	0.9500
O2—C1	1.342 (4)	C9—C10	1.387 (4)
O2—H2	0.86 (4)	C9—H9	0.9500
N1—C2	1.397 (4)	C10—C11	1.385 (5)
N1—C6	1.401 (4)	C10—H10	0.9500
N1—C7	1.448 (4)	C11—C12	1.387 (4)
N2—C20	1.149 (4)	C11—H11	0.9500
N3—C6	1.354 (4)	C12—H12	0.9500
N3—H3A	0.86 (4)	C13—H13A	0.9800
N3—H3B	0.88 (4)	C13—H13B	0.9800
C1—C3	1.368 (4)	C13—H13C	0.9800
C1—C13	1.496 (4)	C14—C19	1.404 (4)
C2—C3	1.437 (4)	C14—C15	1.408 (4)
C3—C4	1.516 (4)	C15—C16	1.382 (4)
C4—C5	1.519 (4)	C16—C17	1.390 (5)
C4—C14	1.538 (4)	C16—H16	0.9500
C4—H4	1.0000	C17—C18	1.382 (5)
C5—C6	1.361 (4)	C17—H17	0.9500
C5—C20	1.421 (4)	C18—C19	1.386 (4)
C7—C8	1.385 (4)	C18—H18	0.9500
			
C1—O2—H2	105 (3)	C8—C9—H9	120.3
C2—N1—C6	121.3 (3)	C11—C10—C9	120.6 (3)
C2—N1—C7	118.7 (2)	C11—C10—H10	119.7
C6—N1—C7	120.0 (2)	C9—C10—H10	119.7
C6—N3—H3A	118 (2)	C10—C11—C12	120.0 (3)
C6—N3—H3B	118 (2)	C10—C11—H11	120.0
H3A—N3—H3B	123 (3)	C12—C11—H11	120.0
O2—C1—C3	122.6 (3)	C11—C12—C7	119.5 (3)
O2—C1—C13	113.5 (3)	C11—C12—H12	120.2
C3—C1—C13	123.8 (3)	C7—C12—H12	120.2
O1—C2—N1	117.1 (3)	C1—C13—H13A	109.5
O1—C2—C3	123.3 (3)	C1—C13—H13B	109.5
N1—C2—C3	119.6 (3)	H13A—C13—H13B	109.5
C1—C3—C2	118.4 (3)	C1—C13—H13C	109.5
C1—C3—C4	119.4 (3)	H13A—C13—H13C	109.5
C2—C3—C4	122.1 (3)	H13B—C13—H13C	109.5
C3—C4—C5	110.7 (2)	C19—C14—C15	114.7 (3)
C3—C4—C14	115.6 (2)	C19—C14—C4	124.6 (3)
C5—C4—C14	108.9 (2)	C15—C14—C4	120.5 (3)
C3—C4—H4	107.1	C16—C15—C14	123.7 (3)
C5—C4—H4	107.1	C16—C15—Cl1	116.4 (3)
C14—C4—H4	107.1	C14—C15—Cl1	120.0 (2)
C6—C5—C20	117.8 (3)	C15—C16—C17	118.7 (3)
C6—C5—C4	123.7 (3)	C15—C16—H16	120.7
C20—C5—C4	118.4 (3)	C17—C16—H16	120.7
N3—C6—C5	123.7 (3)	C18—C17—C16	120.5 (3)
N3—C6—N1	114.8 (3)	C18—C17—H17	119.8
C5—C6—N1	121.4 (3)	C16—C17—H17	119.8
C8—C7—C12	120.6 (3)	C17—C18—C19	119.2 (3)
C8—C7—N1	119.5 (3)	C17—C18—H18	120.4
C12—C7—N1	119.9 (3)	C19—C18—H18	120.4
C7—C8—C9	119.9 (3)	C18—C19—C14	123.2 (3)
C7—C8—H8	120.1	C18—C19—Cl2	115.9 (3)
C9—C8—H8	120.1	C14—C19—Cl2	120.9 (2)
C10—C9—C8	119.5 (3)	N2—C20—C5	179.2 (3)
C10—C9—H9	120.3		
			
C6—N1—C2—O1	−174.8 (3)	C6—N1—C7—C8	−86.5 (4)
C7—N1—C2—O1	3.5 (4)	C2—N1—C7—C12	−86.2 (4)
C6—N1—C2—C3	3.4 (4)	C6—N1—C7—C12	92.2 (3)
C7—N1—C2—C3	−178.2 (3)	C12—C7—C8—C9	−0.4 (5)
O2—C1—C3—C2	−5.5 (5)	N1—C7—C8—C9	178.3 (3)
C13—C1—C3—C2	173.5 (3)	C7—C8—C9—C10	0.5 (5)
O2—C1—C3—C4	177.7 (3)	C8—C9—C10—C11	−0.3 (5)
C13—C1—C3—C4	−3.3 (5)	C9—C10—C11—C12	−0.1 (5)
O1—C2—C3—C1	6.4 (5)	C10—C11—C12—C7	0.3 (5)
N1—C2—C3—C1	−171.7 (3)	C8—C7—C12—C11	0.0 (5)
O1—C2—C3—C4	−176.9 (3)	N1—C7—C12—C11	−178.7 (3)
N1—C2—C3—C4	5.0 (4)	C3—C4—C14—C19	−48.4 (4)
C1—C3—C4—C5	165.1 (3)	C5—C4—C14—C19	76.9 (4)
C2—C3—C4—C5	−11.6 (4)	C3—C4—C14—C15	136.5 (3)
C1—C3—C4—C14	−70.5 (4)	C5—C4—C14—C15	−98.2 (3)
C2—C3—C4—C14	112.8 (3)	C19—C14—C15—C16	−1.0 (4)
C3—C4—C5—C6	11.6 (4)	C4—C14—C15—C16	174.6 (3)
C14—C4—C5—C6	−116.5 (3)	C19—C14—C15—Cl1	179.1 (2)
C3—C4—C5—C20	−169.4 (3)	C4—C14—C15—Cl1	−5.3 (4)
C14—C4—C5—C20	62.5 (4)	C14—C15—C16—C17	1.4 (5)
C20—C5—C6—N3	−1.3 (5)	Cl1—C15—C16—C17	−178.7 (2)
C4—C5—C6—N3	177.7 (3)	C15—C16—C17—C18	0.0 (5)
C20—C5—C6—N1	176.3 (3)	C16—C17—C18—C19	−1.7 (5)
C4—C5—C6—N1	−4.7 (5)	C17—C18—C19—C14	2.2 (5)
C2—N1—C6—N3	174.1 (3)	C17—C18—C19—Cl2	−176.1 (2)
C7—N1—C6—N3	−4.2 (4)	C15—C14—C19—C18	−0.8 (4)
C2—N1—C6—C5	−3.6 (4)	C4—C14—C19—C18	−176.2 (3)
C7—N1—C6—C5	178.0 (3)	C15—C14—C19—Cl2	177.3 (2)
C2—N1—C7—C8	95.1 (3)	C4—C14—C19—Cl2	1.9 (4)

### Hydrogen-bond geometry (Å, º)

*Cg*2 is the centroid of the C7–C12 phenyl ring.

**Table:**

*D*—H···*A*	*D*—H	H···*A*	*D*···*A*	*D*—H···*A*
O2—H2···O1	0.86 (4)	1.72 (4)	2.514 (3)	153 (4)
N3—H3*A*···N2^i^	0.86 (4)	2.22 (4)	3.032 (4)	159 (3)
C16—H16···N2^ii^	0.95	2.62	3.308 (4)	129
N3—H3*B*···*Cg*2	0.88 (4)	2.88 (4)	3.581 (3)	138 (3)
C9—H9···*Cg*2^iii^	0.95	2.70	3.564 (4)	151

Symmetry codes: (i) −*x*+1, −*y*+1, −*z*+2; (ii) −*x*+2, −*y*+1, −*z*+2; (iii) *x*, −*y*−1/2, *z*−1/2.
